# Successful long‐term survival of patients with trisomy 18 and hepatoblastoma after cardiac surgery

**DOI:** 10.1111/ped.70133

**Published:** 2025-09-04

**Authors:** Hideki Tomiyama, Hiroki Hamamoto, Ryo Tanaka, Yoshiro Imai, Koji Komeda, Mitsuhiro Asakuma, Sang‐Woong Lee

**Affiliations:** ^1^ Department of General and Gastroenterological Surgery Osaka Medical and Pharmaceutical University Osaka Japan

**Keywords:** cardiac surgery, hepatoblastoma, malignant neoplasm, survival, trisomy 18

## Abstract

**Background:**

Most patients with trisomy 18 (18T) die of cardiac or other severe anomalies within the first few years. Although surgical intervention for cardiac lesions or other diseases has improved these patients' prognoses in recent years, some survivors face the risk of developing neoplasms, such as hepatoblastoma. However, the treatment strategy remains controversial because of the poor prognosis. We evaluated the merits of aggressive therapeutic interventions for malignant tumors in 18T patients.

**Methods:**

We encountered five patients who underwent hepatoblastoma treatment within the last 11 years. These patients, four girls and one boy, were aged 10 months to 3 years and 9 months at diagnosis; all had previously undergone cardiac interventions.

**Results:**

Three patients were diagnosed with PRETEXT I, whereas the remaining two were diagnosed with PRETEXT II and IV. Primary resection of the tumor was conducted in three patients with PRETEXT I, followed by chemotherapy in two patients. Open biopsy and neoadjuvant chemotherapy were administered in two patients (PRETEXT II and IV), and subsequent tumor resection could be conducted only in the PRETEXT II patient and not in the PRETEXT IV patient. Four patients who underwent liver resection achieved disease‐free survival for 6–10 years postoperatively, and the PRETEXT IV patient did not have tumor regrowth for approximately 2 years and died of another cause. All treatments, including chemotherapy and surgical resection, were administered safely.

**Conclusions:**

Surgical treatment of hepatoblastoma in 18 T patients can be performed safely after cardiac intervention, after obtaining informed consent regarding the associated risks and benefits.

## INTRODUCTION

The prognosis of patients with trisomy 18 (18T) is unfavorable. Most patients die of various severe congenital anomalies within the first few years of life,^1^ with major causes of death including cardiac failure due to malformations.^2^ Although aggressive surgical treatments for patients with 18 T were previously thought to be futile,^3^ surgical cardiac repair and palliative procedures have become more common in recent times, resulting in increased neonatal survival in these infants.^4^ Malignancy is another common prognostic factor, with hepatoblastoma being the most common tumor complication in patients with 18T.^5^

Over the past 11 years, from 2013 to 2023, we encountered five 18T patients with hepatoblastoma who underwent surgical treatment and were followed up for 2–10 years after surgery. Based on the results of these cases and recent literature, we evaluated the merits of aggressive therapeutic interventions for malignant tumors in 18 T patients.

## METHODS

Between 2013 and 2023, 24 patients with 18T were born at or transferred to our hospital for the treatment of various congenital anomalies, including cardiac interventions. Among these patients, we identified those diagnosed with hepatoblastoma during their treatment or follow‐up period. We collected their background information, treatment details, and timing of surgical intervention from their medical records, along with data on their postoperative course. This study was conducted with the informed consent of each affected family member.

## RESULTS

The birth profiles of all the patients are shown in Table 1. Four of the five patients were girls, and one was a boy. These patients were born at a gestational age ranging from 32 weeks to 41 weeks (mean gestational age, 38 weeks), and birthweights ranged from 1160 g to 2240 g (mean weight, 1481 g). All these patients had congenital heart diseases, including Tetralogy of Fallot (TOF) in one patient, ventricular septal defect (VSD) in four patients, patent ductus arteriosus (PDA) in three patients, and atrial septal defect (ASD) (including duplicates) in one patient. Cardiac surgery was performed in all patients, as follows: PDA ligation in three patients (at 1 month of age), pulmonary artery banding in two patients (at 2 and 3 months of age), closure of the VSD in three patients (before the age of 1 year), and radical operation for TOF in one patient (at 3 years of age).

**TABLE 1:** Patients' profile.

Case	Sex	Birthweight(g)	Gestational age	Trisomy	Cardiac anomaly	Cardiac surgery	Other anomalies
1	F	1160	35 weeks 3 days	47, XX, +18	PDA/VSD	PDA ligation PA Banding VSD Closure	None
2	F	2240	41 weeks 1 day	47, XX, +18	TOF	TOF Radical op.	Palatoshisis
3	F	1571	36 weeks 5 days	47, XX, +18	VSD	PA Banding VSD Closure	Syndactyly
4	M	1180	32 weeks 2 days	47, XY, +18	PDA/VSD	PDA Ligation	ARM/UDT
5	F	1257	36 weeks 2 days	47, XX, +18	PDA/ASD/VSD	PDA Ligation AVSD Closure	Palatoschisis

Abbreviations: ARM, Anorectal Malformation; ASD, Atrial Septal Defect; PDA, Patent Ductus Arteriosus; UDT, Undescended Testis; VSD, Ventricular Septal Defect.

Additionally, two patients had palatoschisis, one had syndactyly, and one had anorectal malformation (low‐type, anocutaneous fistula).

The characteristics of the hepatoblastomas are shown in Table 2. The tumors were diagnosed between 11 months and 4 years and 8 months (median: 13 months) of age, and all cases were detected incidentally on CT screening before cardiothoracic surgery. The PRETEXT (PRE‐treatment Tumor EXTension) classification was I in three patients, II in one patient, and IV in one patient. Surgical intervention was performed in all the patients. For the three PRETEXT I patients, subsegmental or partial hepatic resection was performed as the initial treatment, followed by cisplatin (CDDP) chemotherapy in two patients. In the PRETEXT II patient, open biopsy was conducted, and histopathological analysis confirmed the diagnosis. The patient received two courses of CDDP chemotherapy, which resulted in a partial response, allowing for subsequent subsegmentectomy. Postoperatively, two additional courses of the same chemotherapy regimen were administered. The patient with PRETEXT IV also underwent an open biopsy and received four courses of chemotherapy with a combination of CDDP and doxorubicin (DOX). Although the treatment induced a remarkable reduction in tumor size, hepatectomy or chemotherapy was not performed because of the risk of further operation and the patient's general condition.

**TABLE 2:** Summary of characteristics in patients with trisomy 18 and hepatoblastoma.

Case	Age at diagnosis	AFP (ng/mL)	Lesion	PRETEXT	Operation	Outcome	Pathological finding
1	1 year 1 month	33	4	I	Subsegmentectomy	Alive	Mixed epithelial and mesenchymal type
2	4 years 8 moths	28	6, 7	I	Enucleation	Alive	Unknown
3	11 months	501	6	I	Subsegmentectomy	Alive	Epithelial type, Fetal subtype
4	1 year 6 months	98,220	5, 8	II	Open biopsy Subsegmentectomy	Alive	Epithelial type, Fetal subtype
5	10 months	7275	2, 5, 7, 8	IV	Open biopsy	Dead	Epithelial type, Fetal subtype

**FIGURE 1:**
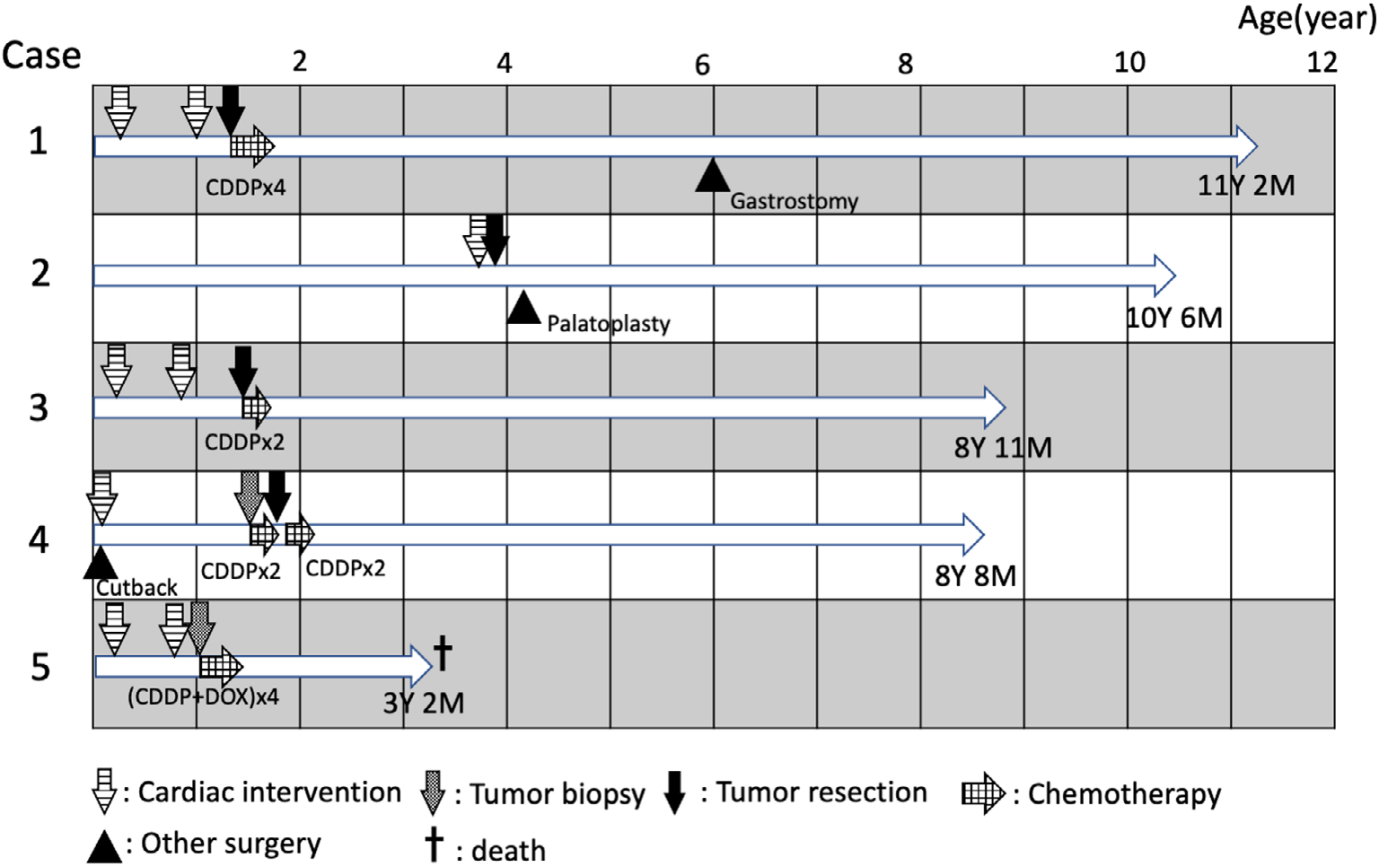
Patients' intervention events and prognoses. Patients in cases 1, 2, 3 (PRETEXT I), and 4 (PRETEXT II) survived beyond 8 years of age, whereas the patient in case 5 (PRETEXT IV) died at the age of 3 years and 2 months.

The entire course of the patient's medical events is shown in Figure 1. During the study period, only one patient (the PRETEXT IV patient) died of acute respiratory failure at 1 year and 6 months after treatment. Regrowth of hepatoblastoma was not observed; however, hepatoblastoma was not directly associated with the cause of death. The remaining four patients survived for 84–120 months.

During the follow‐up period, one patient underwent gastrostomy (Case 1, 6 years old) and another underwent radical operation for palatoschisis (Case 2, 4 years old) for further surgical intervention. Additionally, all surviving patients were diagnosed with epilepsy and began seizure control by medication. However, they were all able to live at home with supportive care in fair to good condition. Periodic CT examination and tumor marker assessment showed no evidence of recurrence.

## DISCUSSION

We have consistently provided aggressive treatment for patients with 18 T and severe congenital heart disease, in accordance with their families' wishes. Among these, we treated five 18 T patients who were diagnosed with hepatoblastoma following cardiac interventions. Their post‐treatment courses were monitored for up to 10 years, allowing us to evaluate the favorable outcomes of their treatments.

18T is one of the most serious genetic disorders, occurring in 1/6000 to 1/8000 live births, with only 5–10% of affected individuals surviving past the first year.^2^ Cardiac diseases due to congenital anomalies are one of the main causes of death. In recent years, however, surgical intervention to correct organ malformations has been applied when appropriate.^6^ As a result, 7.7% of patients now survive for more than 5 years.^7^ Malignant tumors can develop after intervention for congenital heart diseases.^8^ In various malignant tumors, many cases of hepatoblastoma associated with 18 T were frequently reported.^1^, ^8^, ^9^, ^10^, ^11^ Hepatoblastomas are mainly reported in patients aged 3 months to 10 years.^8^ Thus, Farmakis et al. recommended the initiation of a routine tumor surveillance program when choosing to undergo treatment for underlying malformations.^12^ However, implementing such a tumor surveillance program involves a delicate balance of risks and benefits, and no consensus currently exists on recommending this program for all patients with 18 T.^8^ The extent of care in children with 18 T remains a complex and debated decision, involving not only medical but also ethical and psychological considerations.^11^

However, the latest systematic review^8^ reported that 18 T does not preclude resection for hepatoblastoma, and a combination of surgery and chemotherapy should be considered depending on the condition. In recent years, the combination of surgery and chemotherapy has significantly improved the treatment outcomes for hepatoblastoma. Furthermore, the SIOPEL‐3 trial—a randomized comparison of CDDP monotherapy and the conventional CDDP+DOX (PLADO) regimen—demonstrated the non‐inferiority of CDDP monotherapy. This finding established the less toxic CDDP monotherapy as the standard treatment for standard‐risk hepatoblastoma.^13^

In this study, all parents expressed a desire for comprehensive treatment of the liver tumor, in addition to prior surgical interventions for cardiac anomalies. After providing informed consent regarding the potential risks and benefits, they agreed to hepatectomy and/or chemotherapy. As a result, these patients were able to safely undergo treatment despite their cardiac complications. The improvement in their condition following cardiac interventions is believed to have contributed to the observed positive outcomes. These patients achieved far longer prognoses than previously reported,^1^, ^14^ and their extended survival contributed to an enhanced quality of life, leading to greater family satisfaction. These findings suggest that aggressive treatment is both justified and beneficial.

Furthermore, even incomplete treatments—such as those leaving residual tumors or carrying a potential risk of recurrence—were associated with longer prognoses compared to cases without any intervention. For families, prolonging the patient's remaining time through treatment was of significant importance. Therefore, pursuing possible treatment options for high‐risk patients is considered both acceptable and justified.

A limitation of this study is that not all 18 T patients may be treated aggressively for hepatoblastoma. This is because the families of 18 T patients who have undergone extensive cardiac surgery are also aggressive in confronting other life‐threatening diseases compared with families of patients without surgical experience.

In conclusion, although no definitive consensus currently exists regarding radical treatment for hepatoblastoma in 18 T patients, aggressive therapy should be considered to improve the quality of life and prognosis for both patients and their families. However, determining the optimal treatment approach remains a complex decision. The study findings indicate the importance of supporting the patients' lives while respecting the opinions and wishes of their families, who are integral to the child's care journey.

## AUTHOR CONTRIBUTIONS

H. T. designed the study, performed experiments, collected and analyzed data, and wrote the manuscript. H. H, R. T, Y. I, K. K, and M. A. reviewed the manuscript. S. L. reviewed the manuscript and supervised the whole study process. All authors read and approved the final manuscript.

## CONFLICT OF INTEREST STATEMENT

The authors declare no conflicts of interest.
